# Bee Venom Prevents Mucin 5AC Production through Inhibition of AKT and SPDEF Activation in Airway Epithelia Cells

**DOI:** 10.3390/toxins13110773

**Published:** 2021-11-01

**Authors:** Sanga Kim, Hee-Won Kim, Seok-Hwan Chang, Kang-Hyun Leem, Hae-Jeong Park

**Affiliations:** 1 Department of Pharmacology, School of Medicine, Kyung Hee University, 26 Kyungheedae-ro, Dongdae-mun-gu, Seoul 02447, Korea; sanga0568@naver.com; 2 Department of Medical Engineering, Graduate School, Kyung Hee University, 26 Kyungheedae-ro, Dongdae-mun-gu, Seoul 02447, Korea; rlgmldnjs@gmail.com (H.-W.K.); tjrghksekrzj@naver.com (S.-H.C.); 3 Department of Herbology, College of Korean Medicine, Semyung University, 65 Semyung-ro, Jecheon 27136, Korea

**Keywords:** bee venom, asthma, mucus metaplasia, interleukin-13, mucin 5AC

## Abstract

IL-13 induces mucus metaplasia, which causes airway obstruction in asthma. Bee venom (BV) and its components have shown anti-inflammatory effects in allergic diseases such as atopic dermatitis and asthma. In this study, we investigated the effect of BV on IL-13-induced mucus metaplasia through activation of the signal transducer and activator of transcription (STAT6), and regulation of SAM-pointed domain containing Ets-like factor (SPDEF) and forkhead box A2 (FOXA2) in the airway epithelia cell line A549. In A549 cells, BV (1.0 µg/mL) inhibited IL-13 (10 ng/mL)-induced AKT phosphorylation, increase in SPDEF protein expression, and decrease in FOXA2 protein expression—but not STAT6 phosphorylation. BV also prevented the IL-13-induced increase in mucin 5AC (MUC5AC) mRNA and protein expression. Moreover, we observed that inhibition of phosphoinositide 3 kinase (PI3K)/AKT using LY294002 (50 µM) could reverse the alterations in FOXA2 and MUC5AC expression -by IL-13 and BV. However, LY294002 did not affect IL-13- and BV-induced changes in SPDEF expression. These findings indicate that BV inhibits MUC5AC production through the regulation of SPDEF and FOXA2. The inhibition of MUC5AC production through FOXA2 is mediated via the suppression of PI3K/AKT activation by BV. BV may be helpful in the prevention of mucus metaplasia in asthma.

## 1. Introduction

Asthma is a chronic inflammatory disease of the airways. It is characterized by airway hyper-responsiveness (AHR), reversible airflow obstruction and airway remodeling [1]. Airway epithelial cells contribute to asthma reactions, releasing numerous soluble mediators that facilitate the inflammatory response [1]. Th2 cytokines, especially interleukin-13 (IL-13), are involved in the effector phase of the allergic response and promote AHR, mucus overproduction and fibrosis [2]. In the airway epithelial cells, the IL-13 receptor is a heterodimer composed of IL-13Rα1 and IL-4Rα. The binding of IL-13 to its receptor causes activation of the signal transducer and activator of transcription (STAT6), which facilitates mucus metaplasia in asthma [3,4,5].

Mucus in airway epithelium is essential for host defense. However, pathological mucus overproduction contributes to increased asthma morbidity and mortality [6]. In asthma models induced by various stimuli, mucus metaplasia is caused by an increase in production and storage of mucins, particularly mucin 5AC (MUC5AC), in airway epithelial cells [7,8,9]. IL-13 also stimulates MUC5AC production [3,10,11,12] and the IL-13-mediated mucus metaplasia is dependent on the activity of a network of several transcription factors in addition to STAT6 activation. IL-13-induced STAT6 activation increases the expression of the SAM-pointed domain–containing Ets-like factor (SPDEF), which accounts for the initiation of a transcription process in mucus metaplasia [13,14]. SPDEF can suppress transcription factor forkhead box A2 (FOXA2) [13,14], and thus induce mucus metaplasia by up-regulating MUC5AC expression [14,15]. FOXA2 can directly regulate MUC5AC gene transcription as a transcription factor on the MUC5AC gene [16]. In addition, FOXA2 serves in the maintenance of normal differentiation of airway epithelial cells. Thus, the deletion of FOXA2 in mucus cell precursors induces mucus metaplasia, whereas overexpression of FOXA2 inhibits allergen-induced mucus metaplasia [10,16,17]. 

Bee venom (BV), which has been traditionally used in alternative medicine, has been used as an analgesic and immune-modulatory agent for the treatment of chronic inflammatory diseases such as rheumatoid arthritis (RA) and multiple sclerosis [18,19,20]. In addition to BV, experimental studies have shown that its components, such as melittin, apamin and phospholipase A2 (PLA2), have anti-inflammatory properties, inhibiting the production of prostaglandin E2, NO and pro-inflammatory cytokines such as IL-1β, IL-6, tumor necrosis factor-α (TNF-α) and interferon-γ [21,22,23]. Moreover, recent studies have reported that BV and its components could alleviate allergic diseases. BV ameliorates ovalbumin-induced asthma, activating regulatory T cells and reducing the levels of Th2 cytokines [24]. PLA2 derived from BV (bvPLA2) can also modulate regulatory T cells and Th2 cytokine levels [25,26,27]. It ameliorates ovalbumin-induced asthma as well as house dust-mite-induced atopic dermatitis [25,26,27]. Additionally, apamin was shown to have a therapeutic effect on atopic dermatitis. Apamin reduced the activation of NF-κB signaling pathways and suppressed Th2 chemokines and STAT pathways in human keratinocytes cells [28]. Given these effects of BV and its components, BV might modulate Th2 cytokines and/or the STAT pathway, thus exerting a therapeutic effect on asthma. However, to our knowledge, there has been no published report on the effect of BV or its components on mucus metaplasia of asthma to date. In this study, we investigated the protective effect of BV extract—which has been clinically applied, rather than its components [19,29,30]—on mucus metaplasia in asthma, assessing the effect of BV on IL-13-mediated STAT6 activation and MUC5AC production in the human airway epithelial cell line A549. 

## 2. Results

### 2.1. Effect of BV on the Viability of A549 Cells

**Figure 1:**
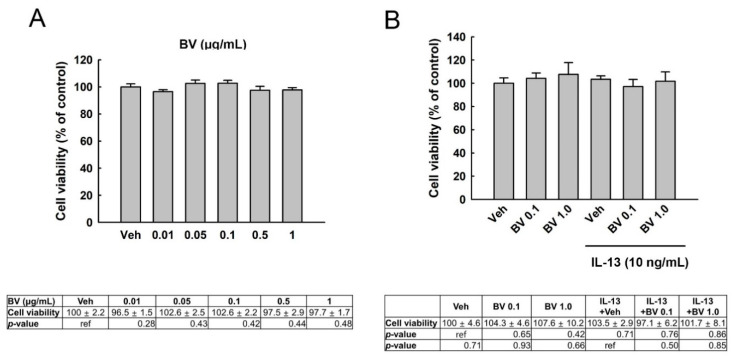
Cytotoxicity of bee venom (BV) in A549 cells. (**A**) A549 cells were treated with various concentrations of BV for 24 h. (**B**) Cytotoxicity of BV was also measured in IL-13-treated cells. BV or vehicle (veh, 1 × PBS) was added to cells, 2 h prior to IL-13 treatment (10 ng/mL, 24 h). Cell viability was determined using an MTT assay. Results are presented as mean ± standard error of the mean (SEM). Independent experiments were performed three times. Ref, reference.

To detect the cytotoxicity of BV on A549 cells, cells were treated with BV at various concentrations (0.01–1 µg/mL) for 24 h (Figure 1A). BV did not induce cytotoxicity at 0.01–1 µg/mL. We also examined the cytotoxicity of BV in IL-13-treated A549 cells. BV (0.1 and 1.0 µg/mL) was added to the cells 2 h prior to IL-13 treatment. Then, IL-13 (10 ng/mL) was treated for 24 h. As shown in Figure 1B, neither BV nor IL-13 induced cytotoxicity in A549 cells. 

### 2.2. Effect of BV on the Phosphorylation of STAT6 in IL-13-Treated A549 Cells

**Figure 2:**
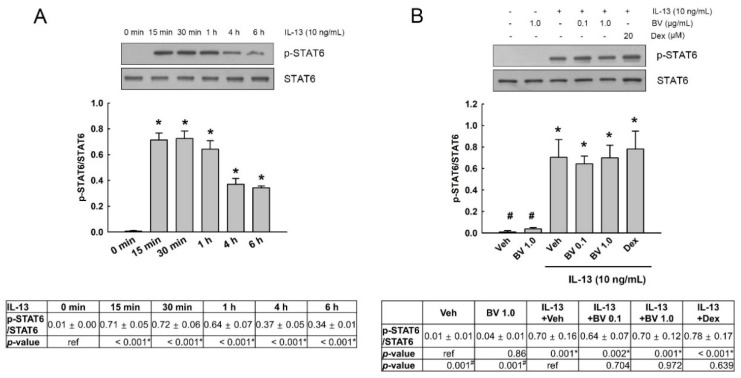
Effect of bee venom (BV) on IL-13-induced signal transducer and activator of transcription (STAT6) phosphorylation. (**A**) Phosphorylation of STAT6 was assessed using Western blot analysis in A549 cells treated with IL-13 (10 ng/mL) for up to 6 h. (**B**) The effect of BV (0.1 and 1.0 µg/mL, 2 h prior to IL-13 treatment) on STAT6 phosphorylation was determined in cells treated with 10 ng/mL IL-13 for 30 min. STAT6 phosphorylation is normalized against its total protein. Data are presented as mean ± standard error of the mean (SEM). Independent experiments were performed four times. One-way ANOVA test, * *p* < 0.05 vs. vehicle (veh) without IL-13; ^#^ *p* < 0.05 vs. veh with IL-13. Ref, reference.

IL-13 binding to its receptor induces the phosphorylation of STAT6, causing AHR and mucus overproduction in asthma [3,4]. In order to assess the alleviating effect of BV on asthma, we examined the effect of BV on the phosphorylation of STAT6 in A549 cells treated with IL-13. First, the phosphorylation of STAT6 by IL-13 was detected in the cells treated with 10 ng/mL IL-13 for up to 6 h. As shown in Figure 2A, IL-13 increased the phosphorylation of STAT6, and peak phosphorylation was observed between 15 min and 1 h. Therefore, we examined the effect of BV on the phosphorylation of STAT6 in cells exposed to IL-13 for 30 min. BV (0.1 and 1.0 µg/mL) and dexamethasone (Dex, 20 µM), which was added as a positive control to compare the effect of BV on IL-13-induced mucus metaplasia, were added 2 h prior to IL-13 treatment. As shown in Figure 2B, BV did not inhibit the IL-13-induced STAT6 phosphorylation at any concentration (0.1 and 1.0 µg/mL; *p* > 0.05 on all). Dex also did not block IL-13-induced STAT6 phosphorylation (*p* > 0.05). 

### 2.3. Effect of BV on the Phosphorylation of Mitogen-Activated Protein Kinases (MAPKs) and AKT in IL-13-Treated A549 Cells

**Figure 3:**
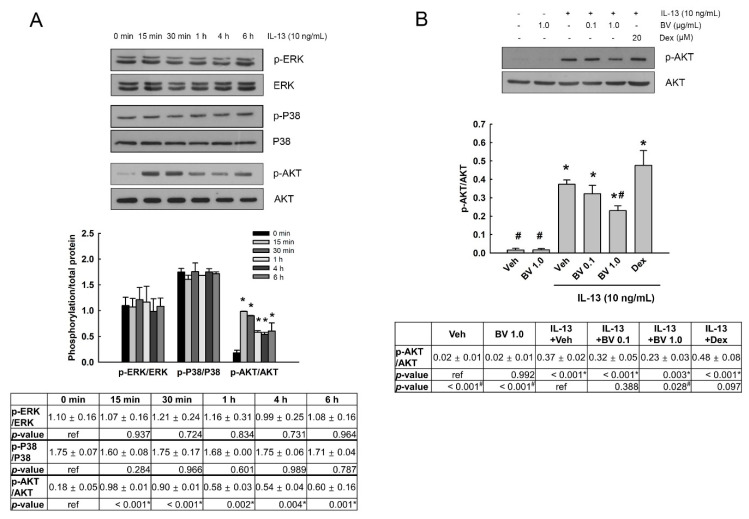
Effect of bee venom (BV) on IL-13-induced AKT phosphorylation. (A) Phosphorylation of mitogen-activated protein kinases (MAPKs) and AKT was examined using Western blot analysis in A549 cells treated with IL-13 (10 ng/mL) for up to 6 h. IL-13 induced ATK phosphorylation, but did not affect phosphorylation of MAPKs. (B) The effect of BV (0.1 and 1.0 µg/mL, 2 h prior to IL-13 treatment) on AKT phosphorylation was determined in cells treated with 10 ng/mL IL-13 for 30 min. Phosphorylation levels are normalized against its total proteins, respectively. Data are shown as mean ± standard error of the mean (SEM). Independent experiments were performed three or four times. One-way ANOVA test, * *p* < 0.05 vs. vehicle (veh) without IL-13; ^#^ *p* < 0.05 vs. veh with IL-13. Ref, reference.

We investigated the effect of BV on the phosphorylation of MAPKs (extracellular signal-regulated kinase (ERK), p38 MAPK (P38) and c-Jun N-terminal kinase (JNK)), and AKT in IL-13-treated A549 cells. First, phosphorylation of ERK, P38, JNK and AKT was detected in the cells exposed to 10 ng/mL IL-13 for up to 6 h. As shown in Figure 3A, IL-13 did not alter the phosphorylation of ERK and P38. Additionally, the phosphorylation of JNK was not detected in IL-13-treated cells (data not shown). In contrast, the phosphorylation of AKT was elevated by IL-13, and peak phosphorylation was observed at 15-30 min. Thus, we examined the effect of BV on the phosphorylation of AKT in the cells exposed to IL-13 for 30 min. BV (0.1 and 1.0 µg/mL) and Dex (20 µM) was added 2 h prior to IL-13 treatment. As shown in Figure 3B, BV at 1.0 µg/mL significantly inhibited IL-13-induced AKT phosphorylation (*p* = 0.028). However, Dex did not suppress IL-13-induced AKT phosphorylation (*p* > 0.05).

### 2.4. Effect of BV on the MUC5AC Expression in IL-13-Treated A549 Cells

**Figure 4:**
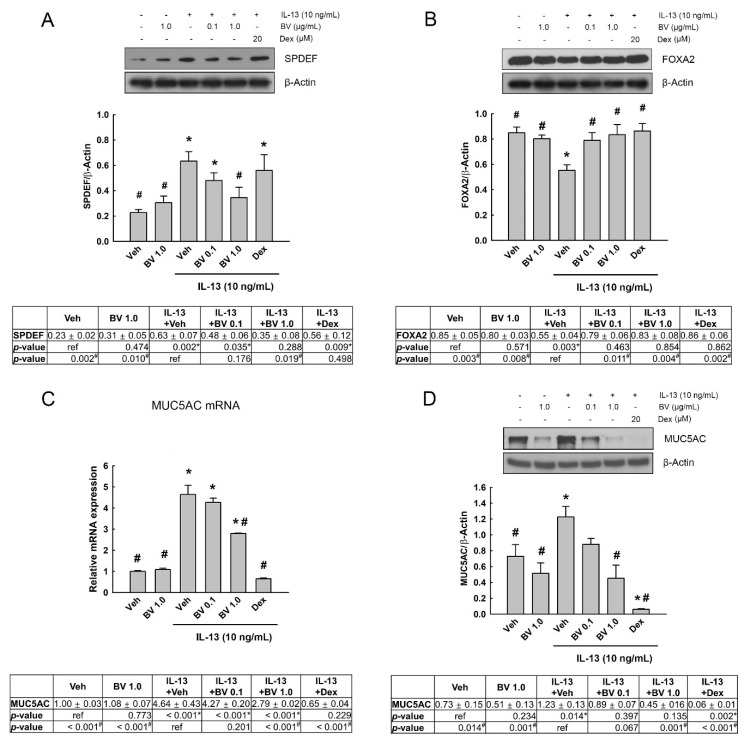
Effect of bee venom (BV) on IL-13-induced mucin 5AC (MUC5AC) expression. The effect of BV on protein expression of SAM-pointed domain containing Ets-like factor (SPDEF) (**A**) and forkhead box A2 (FOXA2) (**B**), which were transcription factors related to MUC5AC expression, was examined in A549 cells using Western blot analysis. (**C**) Quantitative real-time PCR (qRT-PCR) and (**D**) Western blot analysis were also performed in order to determine the effect of BV on mRNA and protein expression of MUC5AC. BV (0.1 and 1.0 µg/mL) was added to cells, 2 prior to IL-13 treatment (10 ng/mL, 24 h). The mRNA and protein expression levels are normalized against GAPDH and β-Actin, respectively. Results are shown as mean ± standard error of the mean (SEM). Independent experiments were performed four times. One-way ANOVA test, * *p* < 0.05 vs. vehicle (veh) without IL-13; ^#^ *p* < 0.05 vs. veh with IL-13. Ref, reference.

Airway mucus overproduction causes airway obstruction and contributes to morbidity and mortality in asthma [6]. IL-13-induced STAT6 phosphorylation promotes MUC5AC overproduction [3,5,31] through the regulation of several transcription factors such as SPDEF and FOXA2 [5,32]. We examined the effect of BV on the expression of SPDEF, FOXA2 and MUC5AC in IL-13-treated A549 cells. BV (0.1 and 1.0 µg/mL) and Dex (20 µM) was added to the cells 2 h prior to IL-13 treatment (10 ng/mL, 24 h). As shown in Figure 4A, IL-13 increased SPDEF expression (*p* = 0.002) and BV at 1.0 µg/mL inhibited the increased expression of SPDEF (*p* = 0.019). In comparison, IL-13 decreased the expression of FOXA2 (*p* = 0.003), and BV suppressed the decrease in FOXA2 expression at both of 0.1 (*p* = 0.011) and 1.0 µg/mL (*p* = 0.004; Figure 4B). Dex inhibited the decrease in FOXA2 by IL-13 (*p* = 0.002), but not the increase in SPDEF expression (Figure 4A,B). Moreover, IL-13 elevated mRNA and protein expression of MUC5AC (*p* < 0.001 and *p* = 0.014, respectively). BV significantly blocked the elevated mRNA and protein expression of MUC5AC at 1.0 µg/mL (*p* < 0.001 and *p* = 0.001, respectively; Figure 4C,D). Dex also suppressed the mRNA and protein expression of MUC5AC (*p* < 0.001 for all).

### 2.5. Effect of Phosphoinositide 3-Kinase (PI3K)/AKT Inhibition on SPDEF, FOXA2 and MUC5AC Expressions in IL-13- and BV-Treated A549 Cells 

**Figure 5:**
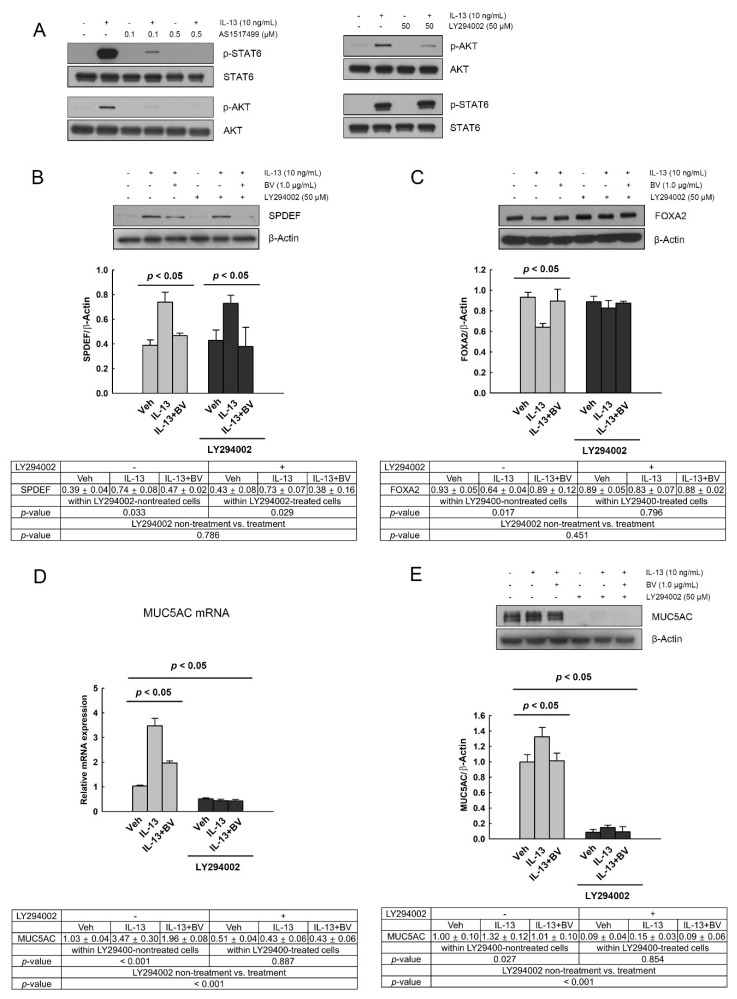
Effect of PI3/AKT inhibition on IL-13- and bee venom (BV)-induced changes in SAM-pointed domain containing Ets-like factor (SPDEF), forkhead box A2 (FOXA2), and mucin 5AC (MUC5AC) expressions. (**A**) The phosphorylation of signal transducer and activator of transcription (STAT6) and AKT was inhibited by STAT6 inhibitor AS1517499 (0.1 and 0.5 µM) and PI3K/AKT inhibitor LY294002 (50 µM) in the IL-13-treated cells, respectively. The effect of LY294002 on SPDEF (**B**) and FOXA2 protein expressions (**C**) was measured in IL-13 and BV-treated in A549 cells using Western blot analysis. (**D**) Quantitative real-time PCR (qRT-PCR) and (**E**) Western blot analysis were also performed in order to detect the effect of LY294002 on IL-13- and BV-induced alteration in MUC5AC mRNA and protein expression. BV (1.0 µg/mL) and LY294002 (50 µM) were added 2 h and 30 min prior to IL-13 treatment (10 ng/mL, 24 h), respectively. The mRNA and protein expression levels are normalized against GAPDH and β-Actin, respectively. Results are mean ± standard error of the mean (SEM). Data were analyzed using two-way ANOVA test. Independent experiments were performed four times.

To investigate whether PI3K/AKT mediated the expression of MUC5AC through SPDEF and FOXA2 in IL-13- and BV-treated A549 cells, we used a specific PI3K/AKT inhibitor. First, we confirmed that the phosphorylation of STAT6 and AKT was inhibited by the STAT6 inhibitor AS1517499 (0.1 and 0.5 µM) and PI3K/AKT inhibitor LY294002 (50 µM) in the IL-13-treated cells, respectively (Figure 5A). Interestingly, AS1517499 inhibited IL-13-induced STAT6 phosphorylation as well as AKT phosphorylation. In contrast, LY294002 could block IL-13-induced AKT phosphorylation, but not STAT6 phosphorylation. This result indicates that AKT phosphorylation is a downstream signaling event of STAT6 phosphorylation in IL-13-treated A549 cells. 

To determine the effect of PI3K/AKT inhibitor on the expressions of SPDEF, FOXA2 and MUC5AC, BV (1.0 µg/mL) and LY294002 (50 µM) were added 2 h and 30 min prior to IL-13 treatment (10 ng/mL, 24 h), respectively. The effect of IL-13 and BV treatment, effect of LY294002, and interaction effect between IL-13 and BV treatment and LY294002 on the expressions of SPDEF, FOXA2 and MUC5AC were analyzed using two-way ANOVA. We found a significant effect of IL-13 and BV treatment on SPDEF expression (*p* = 0.004); whereas LY294002 did not affect SPDEF expression (*p* > 0.05; Figure 5B). An interaction effect between IL-13 and BV treatment and LY294002 was also not observed. We further analyzed the effect of IL-13 and BV treatment within LY29400-non-treated and LY29400-treated cells, respectively, using nested-design two-way analysis. IL-13 and BV treatment significantly changed SPDEF expression in both non-treated (*p* = 0.033) and LY29400-treated cells (*p* = 0.029). This result indicates that the alterations in SPDEF expression by IL-13 and BV are independent of PI3K/AKT activation. 

Figure 5C shows the effect of LY29400 on the FOXA2 expression. In two-way ANOVA, a significant effect of IL-13 and BV treatment on the FOXA2 expression was found (*p* = 0.041), whereas the effect of LY294002 and the interaction effects between IL-13 and BV treatment and LY294002 were not shown (*p* > 0.05). In the analysis of the effects of IL-13 and BV treatment within non-treated and LY29400-treated cells, respectively, we observed that the alteration in FOXA2 expression by IL-13 and BV treatment, shown in non-treated cells (*p* = 0.017), disappeared in LY29400-treated cells (*p* > 0.05). This result means that the alteration of FOXA2 expression in IL-13- and BV-treated cells is mediated by PI3K/AKT activation.

Two-way ANOVA also showed a significant effect of IL-13 and BV treatment on the mRNA expression of MUC5AC (*p* < 0.001), but not on its protein expression (*p* > 0.05). In contrast, the effect of LY294002 was shown to be significant in both mRNA and protein expressions of MUC5AC (*p* < 0.001 on both of them; Figure 5D,E). Interestingly, LY294002 strikingly inhibited MUC5AC mRNA and protein expression. In addition, nested-design two-way analysis revealed that IL-13 and BV treatment could significantly change the MUC5AC mRNA (*p* < 0.001) and protein expression (*p* = 0.027) in non-treated cells (*p* = 0.017), but not in LY29400-treated cells (*p* > 0.05). These findings indicate that MUC5AC mRNA and protein expression is strikingly dependent on PI3K/AKT activation.

## 3. Discussion

In this study, we found a protective effect of BV on mucus metaplasia in asthma. BV inhibited the increase in MUC5AC mRNA and protein expression in IL-13-treated A549 cells, together with a reversal of IL-13-induced alterations in SPDEF and FOXA2 expressions. Interestingly, the change in MUC5AC mRNA and protein expression in IL-13 and BV-treated cells was mediated through PI3/AKT activation. FOXA2 expression was also dependent on PI3/AKT activation, but not SPDEF expression.

STAT6 activation is required for IL-13-induced mucus metaplasia [3]. Our results also showed that IL-13 increased STAT6 phosphorylation as well as MUC5AC mRNA and protein expression in A549 cells. However, BV could inhibit the IL-13-induced increase in MUC5AC expression, but not in STAT6 phosphorylation. Previous studies have reported that the STAT6 and STAT6-linked series of events did not directly regulate MUC5AC transcription [14,33,34]. BV may inhibit MUC5AC expression in mucus metaplasia, not via STAT6, but via other MUC5AC-regulating factors.

IL-13-induced STAT6 phosphorylation activates the transcription factor SPDEF, which can inhibit another transcription factor, FOXA2 [13,14]. Exposure to allergens and cytokines including IL-13 induces a decrease in FOXA2 expression in airway epithelial cells, which has been associated with mucus metaplasia [10,16]. In addition, a previous study showed that FOXA2 directly regulated MUC5AC expression as a transcription factor on the MUC5AC gene [16]. In our study, we found that BV suppressed the IL-13-induced elevation in SPDEF and reduction in FOXA2. BV may inhibit MUC5AC expression in mucus metaplasia via the regulation of SPDEF and FOXA2.

**Figure 6:**
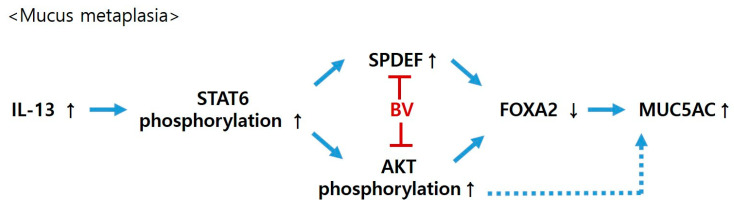
Possible protective mechanism of bee venom (BV) against mucus metaplasia. IL-13, interleukin 13; STAT6, signal transducer and activator of transcription; SPDEF, SAM-pointed domain containing Ets-like factor; FOXA2, forkhead box A2; MUC5AC, mucin 5AC.

Moreover, BV was able to prevent IL-13-induced AKT phosphorylation. We also observed that the STAT6 inhibitor AS1517499 could block IL-13-induced STAT6 and AKT phosphorylation, whereas the PI3K/AKT inhibitor LY294002 suppressed AKT phosphorylation but not STAT6 phosphorylation. This result indicates that AKT phosphorylation is a downstream signaling event of STAT6 phosphorylation, and is mediated by the activation of STAT6 in mucus metaplasia. In addition, previous studies have shown that LY294002 could block the IL-13-induced increase in MUC5AC expression and mucus metaplasia [9,12,35]. Consistently with these previous reports, we found that LY294002 obstructed the change in FOXA2 and MUC5AC expressions shown in IL-13- and BV-treated cells. In contrast, LY294002 did not affect the alteration in SPDEF expression by IL-13- and BV. These findings indicate that BV may prevent IL-13-induced FOXA2-MUC5AC production in A549 cells through the regulation of two molecules—the inhibition of AKT phosphorylation, and SPDEF activation (Figure 6).

Intriguingly, LY294002 more strikingly affects the block of MUC5AC mRNA and protein expression than FOXA2 expression. The expression levels of MUC5AC in LY294002-teated cells was lower than vehicle-treated cells without IL-13 treatment. Tyner et al. [9] reported that PI3K/AKT participated in IL-13-induced MUC5AC expression and mucus metaplasia through the inhibition of the apoptosis of ciliated airway epithelial cells. Yan et al. [35] showed that PI3K contributed to IL-13-induced MUC5AC expression through activation of the transcription factor NFAT3. In our study, PI3K/AKT was involved in IL-13-induced MUC5AC expression via the regulation of FOXA2. These findings indicate that PI3K/AKT may regulate IL-13-stimulated mucus production via various molecular mechanisms. Therefore, considering that BV could inhibit IL-13-induced AKT phosphorylation, BV may prevent MUC5AC expression in mucus metaplasia, not only through AKT-dependent FOXA2, as shown in our results, but also through other AKT-dependent molecules.

BV can induce allergenic reactions and anaphylactic responses [36]. A component of BV, bvPLA2, is considered a major inflammatory trigger and a main allergenic substance of BV [37]. Nevertheless, studies have reported that BV and bvPLA2 exert anti-inflammatory effects in allergic diseases such as atopic dermatitis and asthma. BV alleviated atopic dermatitis through the inactivation of the complement system [38]. bvPLA2 relieved skin lesions in atopic dermatitis murine models, together with the inhibition of serum immunoglobulin E (IgE) and cytokine levels, and macrophage and mast cell infiltration [26,27]. In ovalbumin-induced asthma mice, BV and bvPLA also activated regulatory T cells and reduced the levels of Th2 cytokines and infiltration of inflammatory cells in the bronchoalveolar lavage fluid [24,25]. To determine whether bvPLA2 induced inflammation and allergenic reactions, further experimental and clinical studies may be needed. Even if bvPLA2 induces inflammation and allergenic reactions, we expect that BV may be useful for the alleviation of asthma. A previous study showed a significant anti-inflammatory effect of BV in RA mice [39]. The authors suggested that a major component of BV, melittin, could form a melittin–bvPLA2 complex and, thus, that melittin inhibited the proinflammatory activity of bvPLA2 [18,39]. In addition, we suggest that BV may be helpful as a combination drug in inhaled corticosteroid therapy, which is a major maintenance therapy for asthma. Corticosteroids have an inhibitory effect on PLA2, which is one of the anti-inflammatory mechanisms suppressing the synthesis of prostaglandins. The effect of Dex, shown in our study, also supports the validity of the combination of BV and corticosteroids. Dex could potently inhibit the increase in MUC5AC expression and the decrease in FOXA2 expression induced by IL-13. However, it did not block the increase in AKT phosphorylation and SPDEF expression, in contrast to BV. These results indicate that Dex may inhibit FOXA2-MUC5AC production, not through AKT or SPDEF activation, but through the regulation of other molecules. Given that, the addition of BV to inhaled corticosteroid therapy may exert a synergistic effect in the maintenance therapy of asthma.

Clinically and experimentally, BV has been administrated by direct sting of the bee, injection, or acupuncture (also called apitherapy). Most clinical studies and practices use apitherapy, which is performed by subcutaneous injection of diluted BV into an acupoint [30,40,41,42]. BV apitherapy and injection have shown significant therapeutic effects in Parkinson’s disease, osteoarthritis, and pain relief [30,40,41,42,43]. Although no clinical trial of BV administration has been reported on respiratory diseases, including asthma, animal studies have shown that BV injection (via acupoint or intraperitoneally) can alleviate pleurisy and asthma [24,44]. Thus, we expect that BV injection (subcutaneously) may be also a proper rout for the maintenance therapy of asthma. Recently, Park et al. [45] reported that in vitro release of BV from BV-loaded poly-(lactic-co-glycolic acid; PLGA) particles, which have been broadly studied as a drug delivery carrier of proteins and peptides, was sustained over 1 month. Delivery carriers of BV would help in the maintenance therapy of asthma by reducing the frequency of BV injections. Moreover, a study using pig showed that administration of chitosan/alginate nanoparticle-encapsulated BV via the nasal route facilitated systemic immune response and improved viral clearance in the infection of porcine reproductive and respiratory syndrome virus (PRRSV), increasing levels of PRRSV-specific IgG and viral neutralizing antibody in the serum and tissues (lung and bronchial lymph node) [46]. This indicates that the topical application of BV may be also possible. However, there are some limitations in BV injection for asthma treatment. BV may exert its activity not only in the respiratory system, but also systemically. In atopic dermatitis mice, subcutaneous BV injection decreased the activity of the complement system in lesioned skin as well as in serum [38]. In RA rats, subcutaneous BV injection reduced pro-inflammatory cytokine levels in the plasma [39]. Nasal administration of chitosan/alginate nanoparticle-encapsulated BV also showed a systemic immune response [46]. For asthma treatment, a respiratory-targeted response rather than a systemic response may be desirable, such as with inhaled corticosteroids. Future clinical and experimental studies for respiratory-targeted response of BV are needed. Additionally, BV should be used only in asthma patients with a negative response to BV in BV allergy tests.

In our study, there are some limitations. Our study only employs a cell culture study using the A549 cell line, without any animal or clinical study. We only focus on the expression of MUC5AC and its upstream signaling pathway. To support our conclusion, the effect of BV needs to be examined in asthma animal models or patients with asthma. Further studies using three-dimensional cultures that simulate the pathophysiological microenvironment more closely, such as an air–liquid interface culture, would be also helpful in confirming mucin production. In addition, we do not address the effects of each component of BV. The effects of each component of BV on asthma need to be clarified in further studies. In particular, we expect that the melittin or apamin may reveal potential alleviating effects on asthma, considering their significant anti-inflammatory activities shown in previous studies [21,22,23]. The effect of bvPLA2 should be also studied in its various concentrations to clarify whether bvPLA2 is helpful in relieving or if it worsens asthma.

## 4. Conclusions

In conclusion, BV prevented the increase in MUC5AC expression in IL-13-treated A549 cells through the inhibition of the elevation of SPDEF and reductions in FOXA2. FOXA2 and MUC5AC expressions, but not SPDEF expression, were mediated by PI3K/ATK activation in IL-13-treated A549 cells. BV blocked IL-13-stimuated AKT phosphorylation. Taken together, BV may prevent FOXA2-regulating MUC5AC production through the inhibition of AKT activation, as well as through the suppression of enhanced SPDEF in airway epithelial cells. These results suggest that BV has a protective effect against asthma, inhibiting mucous metaplasia.

## 5. Materials and Methods

### 5.1. Cell Culture and Treatment

The human airway epithelial cell line A549 was supplied by the Korean Cell Line Bank (Seoul, Korea). Cells were cultured in Dulbecco’s modified Eagle’s medium supplemented with 10% fetal bovine serum and 100 U/mL penicillin/streptomycin. Cells were maintained in a humidified incubator at 37 °C in an atmosphere containing 5% CO_2_. The cell medium was changed every 2 days.

Recombinant human IL-13 (R & D Systems, Minneapolis, MN, USA) was dissolved in 1 × PBS and was added to A549 cells at a dose of 10 ng/ml. Dried whole BV from Apis mellifera (honeybee) was purchased from Sigma-Aldrich (St. Louis, MO, USA) and prepared fresh in 1 × PBS. To evaluate the effect of BV on IL-13-treated cells, the cells were incubated with BV (0.1 and 1.0 µg/mL) or vehicle (1 × PBS) 2 h prior to IL-13 treatment.

### 5.2. Cell Viability Assay

To assess the cytotoxicity of BV, a 3-(4,5-dimethylthiazol-2-yl)-2,5-diphenyltetrazolium bromide (MTT) assay was performed. After cells were treated with BV and with or without IL-13, MTT (Sigma-Aldrich) solution was added to the cells for 4 h. Then, the cells were incubated with MTT-solubilizing solution for 1 h. To determine viability, absorbance was measured at a test wavelength of 570 nm using a microplate reader (Molecular Devices, Toronto, ON, Canada).

### 5.3. Western Blot Analysis

Cells were lysed using CelLytic M buffer (Sigma-Aldrich) containing a 1 × protease/phosphatase inhibitor cocktail (Cell Signaling Technology, Beverly, MA, USA). The protein concentration was assessed using Bradford reagent (Sigma-Aldrich). Equal amounts of protein (50 µg) were separated on sodium dodecyl sulfate (SDS)-polyacrylamide gels, and transferred onto a nitrocellulose membranes (Amersham Biosciences, Uppsala, Sweden). After blocking with 5% skimmed milk, the membranes were incubated with rabbit phospho-STAT6, rabbit phospho-ERK, rabbit phospho-P38, rabbit phospho-JNK, rabbit phospho-AKT, rabbit FOXA2 (Cell Signaling Technology), rabbit SPDEF (Abcam, Cambridge, UK) and mouse MUC5AC antibodies (Merck Millipore, Darmstadt, Germany), overnight at 4 °C. Then, these were further incubated with horseradish peroxidase-conjugated anti-mouse or anti-rabbit IgG (GeneTex, Irvine, CA, USA). For band detection, an enhanced chemiluminescence (ECL) substrate (Bio-Rad Laboratories, Hercules, CA, USA) was used. The band density was quantified using ImageJ software (NIH).

### 5.4. Quantitative Real-Time PCR (qRT-PCR)

Total RNA was extracted from the cells using an RNeasy Mini kit (Qiagen, Hilden, Germany). cDNA was synthesized from total RNA using a DiaStar RT Kit (SolGent, Daejeon, Korea) according to the manufacturer’s instructions. qRT-PCR was conducted using Real-Time PCR EvaGreen Kit (SolGent) with specific primers on MUC5AC gene (5’-AGAATCTCTGGTCCTGAA-3’; 5’-GGTTGTGCTAGTTGTAGA-3’). The analysis was performed using a StepOnePlus Real-Time PCR System (Applied Biosystems Inc., Carlsbad, CA, USA). The relative quantification of MUC5AC mRNA was calculated based on the 2^−ΔΔ^CT method [47]. The expression level of GAPDH mRNA as a housekeeping gene was used to analyze the relative expression level of MUC5AC mRNA [48].

### 5.5. Statistical Analysis

Data are expressed as means ± standard errors of the mean (SEMs). Statistical analysis was performed using IBM SPSS Statistics 23 (SPSS Inc., Chicago, IL, USA). The normality of variable distribution on the data was confirmed by the Shapiro–Wilk test. All the data followed a normal distribution, with the homogeneity of variances verified by the Levene test. The effects of BV and IL-13 on cytotoxicity, phosphorylation levels, and expression at the mRNA and protein levels were evaluated by one-way analysis of variance (ANOVA), followed by the LSD post-hoc test. To assess whether the expression levels of SPDEF, FOXA2 and MUC5AC were affected by LY294002 in IL-13- and BV-treated cells, two-way ANOVA was performed. Values of *p* < 0.05 were considered statistically significant.

## Data Availability

Not applicable.
